# Importance of circadian timing for aging and longevity

**DOI:** 10.1038/s41467-021-22922-6

**Published:** 2021-05-17

**Authors:** Victoria A. Acosta-Rodríguez, Filipa Rijo-Ferreira, Carla B. Green, Joseph S. Takahashi

**Affiliations:** 1 Department of Neuroscience, Peter O’Donnell Jr. Brain Institute, University of Texas Southwestern Medical Center, Dallas, TX USA; 2 Howard Hughes Medical Institute, University of Texas Southwestern Medical Center, Dallas, TX USA

**Keywords:** Circadian rhythms and sleep, Ageing, Metabolism, Feeding behaviour

## Abstract

Dietary restriction (DR) decreases body weight, improves health, and extends lifespan. DR can be achieved by controlling how much and/or when food is provided, as well as by adjusting nutritional composition. Because these factors are often combined during DR, it is unclear which are necessary for beneficial effects. Several drugs have been utilized that target nutrient-sensing gene pathways, many of which change expression throughout the day, suggesting that the timing of drug administration is critical. Here, we discuss how dietary and pharmacological interventions promote a healthy lifespan by influencing energy intake and circadian rhythms.

## Abstract (web summary)

Circadian clocks link physiologic processes to environmental conditions and a mismatch between internal and external rhythms has negative effects on organismal health. In this review, the authors discuss the interactions between circadian clocks and dietary interventions targeted to promote healthy aging.

## Introduction

Aging is a major risk factor for chronic diseases, including obesity, diabetes, cancer, cardiovascular disease, and neurodegenerative disorders^1^. Improvements in healthcare have increased life expectancy worldwide, but as the aged population increases, frailty and morbidity have become a public health burden. Through the Healthy Life Expectancy (HALE) indicator, the World Health Organization estimates that worldwide humans spend >10% of our lives suffering from age-related diseases. Aging research currently focuses on closing the gap between lifespan (living longer) and healthspan (remaining healthier for longer). While lifespan can be easily determined with survival curves, healthspan is more complex to quantify. Several biomarkers of healthspan are used in animal models^2,3^ and humans^4^, ranging from levels of metabolites (glucose, cholesterol, fatty acids), biological processes (inflammation, autophagy, senescence, blood pressure) to biological functions (behavior, cognition, cardiovascular performance, fitness, and frailty).

A major challenge is to understand, mechanistically, the progressive deregulation of metabolic function and to design interventions to delay the onset of age-related diseases. Research in animal models has revealed that the aging process can be targeted using genetic, nutritional, and pharmacological interventions^5^.

Dietary restriction widely improves lifespan and healthspan^1^, and some of its benefits could be mediated by the circadian system. Interactions between circadian clocks and aging-related pathways, such as the sirtuin^6–8^, insulin/IGF-1^9^, and mTOR^10^ signaling pathways, support this hypothesis; yet, the mechanisms are not fully understood. Here, we review the literature from animal models and human trials assessing the effects of feeding paradigms and food quality on circadian rhythms, health, and lifespan. Finally, we discuss concepts of circadian medicine as an opportunity for tailoring antiaging interventions.

## Circadian rhythms and aging

In mammals, the circadian system controls daily (~24 h) rhythms in behavior and physiology^11^. This evolutionarily conserved timekeeping mechanism allows organisms to synchronize internal processes with environmental timing cues, ensuring optimal organismal adaptation^12^.

**Fig. 1:**
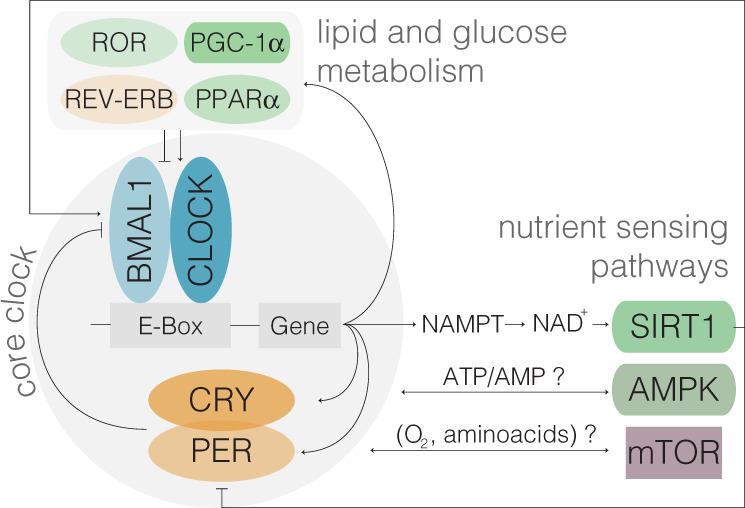
Crosstalk between molecular components of circadian clock, nutrient-sensing, and metabolic pathways. Core clock proteins CLOCK/BMAL1 (transcriptional activators) and PER/CRY (repressors) are engaged in an autoregulatory transcriptional/translational feedback loop leading to 24 h oscillations in gene expression, activity and protein levels. The molecular clock also regulates the rhythmic expression of genes involved in several cellular functions and nutrient-sensing pathways, which in turn feedback to the core clock machinery. CLOCK (*Clock*), BMAL1 (*Arntl*), PER (*Period*), CRY (*Cryptochrome*), SIRT1 (Sirtuin 1), AMPK (5’ AMP-activated protein kinase), PGC-1α (PPARγ co-activator 1a), mTOR (Mammalian target of rapamycin), ROR (RAR- Related Orphan Receptor), Rev-Erb (*Nr1d1*), and PPAR (Peroxisome Proliferator Activated Receptor).

**Fig. 2:**
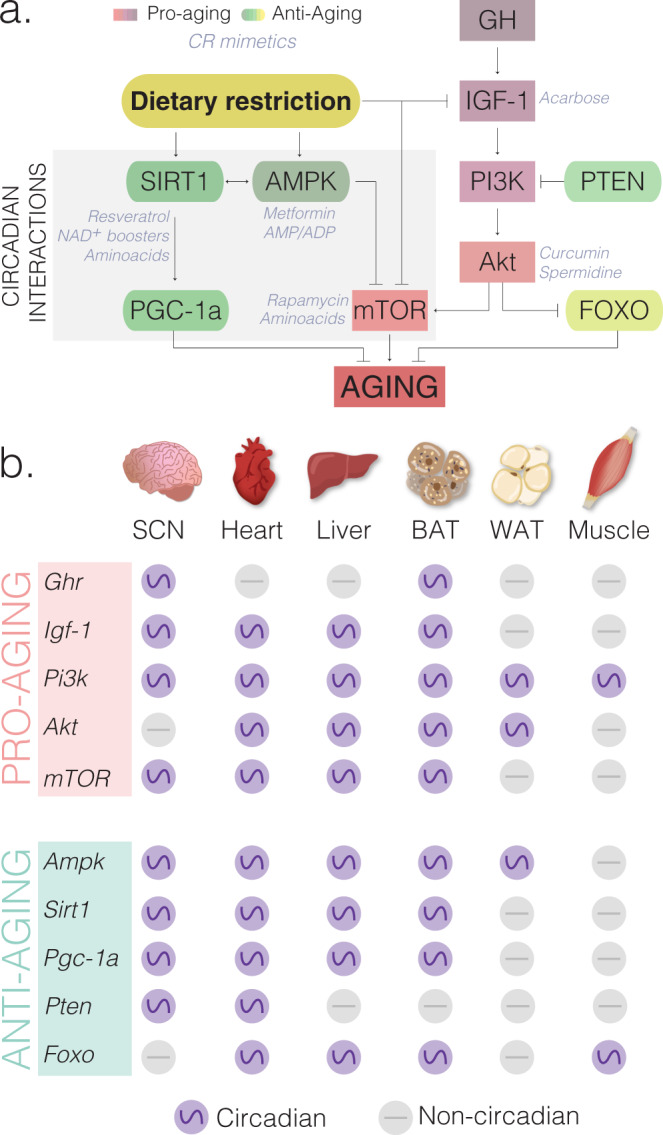
Aging-related pathways regulated by dietary interventions display circadian oscillations. **a** Dietary interventions influence nutrient-sensing pathways^50^ by inducing antiaging (yellow-green gradient) and reducing proaging (pink-purple gradient) molecules. There are known direct interactions between nutrient-sensing pathways involve in aging (SIRT1, AMPK, NAMPT, mTOR) and core clock genes (CLOCK/BMAL and PER/CRY). Several compounds known as caloric restriction mimetics (CRM, shown as gray font color) target specific nutrient-sensing genes and mimic the health benefits of CR without reducing food intake. CRMs tested by ITP and other laboratories include acarbose, curcumin, spermidine, rapamycin, metformin, resveratrol, NAD+ boosters and amino acids (reviewed by ref. ^172^). CRMs (gray font color) are indicated next to or below their molecular targets. **b** Aging-related genes are expressed in a circadian manner (purple circles) in at least one tissue. Genes that are noncircadian in each tissue are represented as gray circles. RNA-seq and microarray containing circadian datasets for liver^16^ and other tissues extracted from CircaDB (http://circadb.hogeneschlab.org, Hogenesch laboratory). Proaging molecules: GH (growth hormone), GHR (growth hormone receptor), IGF-1 (insulin-like growth factor 1), PI3K (Phosphoinositide 3-kinase), AKT (also known as Protein kinase B), and mTOR (Mechanistic or Mammalian target of rapamycin). Antiaging molecules: SIRT1 (Sirtuin 1), AMPK (5’ AMP-activated protein kinase), PGC-1α (PPARγ co-activator 1a), PTEN (Phosphatase and tensin homolog), FOXO (Forkhead Box O). Core clock components CLOCK/BMAL1 (transcriptional activators) and PER/CRY (transcriptional repressors). See also Supplementary Table 1 for additional tissues and aging-related genes in mouse, baboon, and humans.

The central clock, located in the hypothalamic suprachiasmatic nucleus (SCN), synchronizes peripheral clocks in tissues throughout the body via humoral and neural signals^13^. Peripheral clocks are cell-autonomous, and while synchronized by the central clock, do not require SCN inputs to generate rhythms. Rather, they are composed of transcriptional/translational negative feedback loops of transcriptional activators (CLOCK/BMAL1) and repressors (PER/CRY) driving their own oscillations^14^ and regulating the rhythmic expression of genes involved in key cellular functions^15,16^ (Fig. 1). Glucose, fatty acid, and cholesterol metabolic pathways are under circadian control, and the disruption of clock genes alters metabolism and worsens health status^11^. Moreover, components of nutrient-sensing pathways associated with aging exhibit tissue-specific oscillations due to a direct crosstalk with core clock genes^15^ (Figs. 1–2, see Caloric Restriction below).

The endocrine system is also regulated in a circadian manner^17^. In humans, insulin, ghrelin, adiponectin, and cortisol are elevated in the morning/afternoon, while melatonin, thyroid stimulating hormone, prolactin, leptin, growth hormone (GH), and fibroblast growth factor 21 (FGF-21), are elevated in the evening/night^17^. These rhythmic hormones regulate feeding and sleep, and also synchronize endogenous clocks^18^. Hormonal rhythms may be central to coordinate internal clocks during aging, as timed-administration of insulin, corticosterone, and prolactin mimicking the rhythmic pattern seen in young rats, is sufficient to reverse insulin resistance and reduce body fat in aged rats^19^. Circadian clocks can regulate metabolic and endocrine rhythms independently of feeding and sleep^20,21^. For example, while nutrients account for circulating glucose during feeding events, the liver clock provides the glucose required during the fasting phase, leading to nearly constant levels of glucose throughout the day^20^. Interestingly, aspects of aging are reversed in parabiosis experiments, in which the circulatory systems of aged and young mice are connected^22^. Although possible, whether humoral rhythms from young mice contribute to aging reversal is unknown.

The systemic influence of circadian rhythms on tissue homeostasis, sleep regulation, and behavior is well established; with direct links to aging^23,24^. High-amplitude circadian rhythms correlate with wellbeing and increased lifespan in animal models regardless of food composition^25–27^, while circadian rhythms decrease in amplitude with normal aging and often exhibit a shift in phase^23,28^. Additionally, aged animals have defects in entrainment/synchronization to light/dark cycles^8,29–31^, which impairs the ability of the organism to predict and adapt to environmental changes.

A mismatch between internal clocks and daily changes in the environment is detrimental to survival. Mice with free-running periods of ~24 h live 20% longer than mice whose periods deviate significantly from 24h^32^. Conversely, mice with internal period of ~24 h reduce their lifespan when exposed to a short day of 4 h light/4 h dark as compared to a 24 h day (12 h light/12 h dark)^33^. Furthermore, rodents deficient in clock genes have shortened lifespans, including *Clock*^*−/−*^ *mice*^25^, *Bmal1*^*−/−*^ mice^34^, and 20-h-period golden hamsters carrying a *tau* mutation^35^. Remarkably, interventions that restore proper circadian rhythmicity improve longevity. For instance, transplantation of fetal SCN into aged animals increases rhythmicity and extends lifespan^35–37^. Conversely, genetic perturbation of circadian genes in peripheral tissues in rodents is associated with metabolic disorders^20,38–41^. Disruption of the clock through lifestyle (i.e., jet lag, shift work) is associated with decreased lifespan in mice^42^ as well as increased risk of cancer^43^, cardiovascular disease, and metabolic disruption in humans^44^. How aging perturbs the function of internal clocks remains an open question, but collectively these data suggest that understanding the circadian regulation of physiology and metabolism may provide novel insights for designing and implementing antiaging interventions.

## Feeding paradigms in animal models

**Fig. 3:**
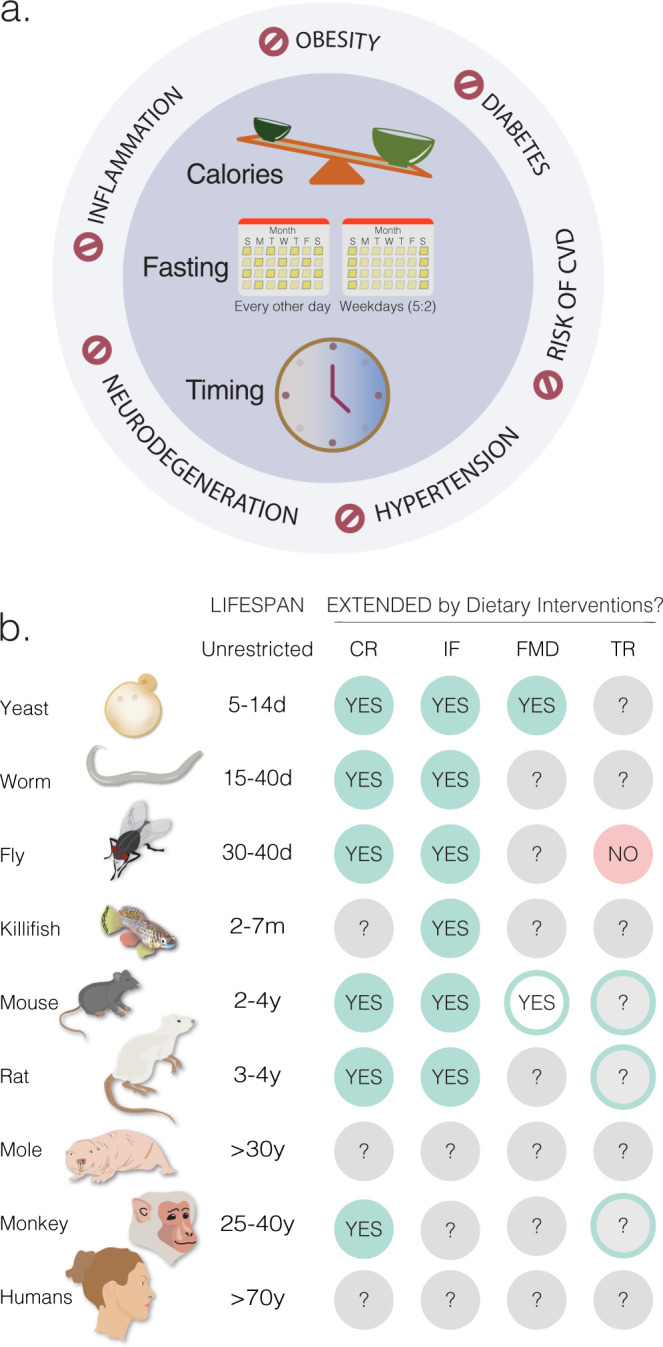
Dietary interventions protect against chronic diseases and promote lifespan. **a** Dietary restriction improves healthspan in several species by reducing the risk for obesity, diabetes, cardiovascular disease (CVD), hypertension, neurodegeneration, and inflammation. Although these benefits have been associated with a reduction in the number of calories consumed, extending fasting periods, and restricting the timing of food intake, the individual contribution of these factors remains unknown, since classical dietary restriction protocols often combines one or more of these parameters. **b** Dietary restriction extends lifespan in most model organisms used in aging research. Green circles labeled as “YES” represent dietary interventions that increase both maximum and median lifespan. Green circles with white centers labeled as “YES” represent the extension of median but not maximum lifespan. Red circles labeled as “NO” refer interventions that have no effect on median or maximum lifespan. Gray circles labeled as “?” indicate conditions that have not yet been tested. Although time-restricted feeding (TR) does not extend lifespan in flies^204^, caloric restriction (CR) protocols that promote longevity in monkeys, rats and mice also involve TR as well (shown as green outer circles with gray centers labeled with “?”). Dietary interventions: Caloric Restriction (CR)^27,77,205,206^. Intermittent Fasting (IF)^92,207^ includes either periodic fasting (PF, also known as every-other-day feeding EOD) or 5 days fasting/low calories followed by 2 days unrestricted intake (Weekdays 5:2). Fasting Mimicking Diet (FMD)^100^ with 5 days of a low-caloric diet every 3–4 months. Time-restricted feeding (TR)^204^, in which food is available ~8–12 h exclusively during the active period.

In the last two decades we have come to appreciate that, in addition to caloric content, timing of feeding and fasting periods between meals influence wellbeing^45,46^. Recent findings highlight the influence of dietary restriction (DR) on circadian clocks, yet the mechanisms linking circadian systems with metabolism, epigenetic signatures, and age-related diseases remain to be elucidated. Furthermore, the role of peripheral clocks in regulating metabolic homeostasis during aging is still unclear. Understanding the mechanistic links between circadian regulation during dietary interventions is an exciting avenue that may provide insights on how to achieve optimal health benefits throughout life. Here we discuss how health and lifespan are influenced by the most commonly used DR paradigms: caloric restriction (CR), time restriction (TR), and intermittent fasting (IF), as well as changes in dietary composition, in animal models (Fig. 3).

### Caloric restriction

CR extends lifespan and improves health in several organisms^1^. CR reduces oxidative stress, increases insulin sensitivity, modulates neuroendocrine responses, impacts central nervous system function^47^, reduces necroptosis^48^, and delays the onset of neoplasia^49^. Overall, CR ameliorates several hallmarks of aging, including deregulated nutrient-sensing pathways, mitochondrial dysfunction, impaired autophagy, stem cell senescence, DNA damage, and inflammation^1^.

CR promotes longevity by targeting highly conserved pathways across species^50^. These include inhibition of IGF-1 and mTOR as well as the activation of SIRT1, NAMPT, AMPK, FGF-21, and PGC-1α signaling pathways (Fig. 2a). These molecules exhibit tissue-specific circadian oscillations (Fig. 2b and Supplementary Table 1), and some of them feedback to molecular components of the clock^14,15,23^ (Fig. 1). For instance, CLOCK-BMAL1 rhythmically activate NAMPT, the rate-limiting enzyme in NAD+ biosynthesis, leading to circadian oscillations of NAD+ levels^7^. As a NAD+-dependent deacetylase, SIRT1 directly binds to CLOCK-BMAL1 and rhythmically deacetylates and promotes degradation of PER2, contributing to the maintenance of robust circadian rhythms^7,51^. Furthermore, AMPK, an energy sensor of AMP/ATP ratios, destabilizes the circadian transcriptional repressor CRY1^52^.

Inhibition of the insulin/IGF-1 signaling pathway increases lifespan from *C. elegans*^53^ to mice^54^, and polymorphisms in this pathway have been found in centenarians^55^. Intriguingly, IGF-1 levels decline with both normal and premature aging, perhaps as a defensive response to energy scarcity^56^. *Igf-1* expression oscillates in mouse tissues, including the liver, where most IGF-1 is produced^57^. Additionally, IGF-1 rhythms in the liver and blood are abolished in mice lacking core clock genes (*Cry1/2*^−/−^ mice)^9^. Downstream targets of IGF-1 also display tissue-specific oscillations, suggesting that IGF-1 signaling throughout the body is under circadian control^57^. IGF-1 may be one of signals that synchronizes peripheral clocks to feeding time^58^; however, circadian regulation of the IGF-1 pathway throughout the lifespan has been largely unexplored.

mTOR plays a critical role in energy metabolism, and regulates circadian period in flies^59^ and mice^60^. It has been implicated in the entrainment of the circadian clock in the mouse brain^61^ and the activity of the mTOR complex oscillates in several brain regions and in peripheral tissues^60^. CR restores the rhythmic activity of mTOR that is lost under ad lib feeding in arrhythmic mutant mice^62^, perhaps through self-imposed feeding/fasting cycles associated with CR protocols^63^. Moreover, mTOR inhibition upon fasting is driven by induction of the core clock protein PER2^64^. These studies indicate that both endogenous clocks and CR regulate the rhythmic activity of mTOR.

A genome-wide circadian analysis of liver^65^ and epithelial stem cells^66^ showed that CR rescues the age-related decline in circadian metabolic pathways in mice. These findings suggest that the aging process has minimal effect on core clock machinery, and instead promotes tissue-specific reprogramming of the transcriptome. Interestingly, BMAL1-deficient mice not only have reduced lifespan but also exhibit premature aging phenotypes such as sarcopenia, cataracts, less subcutaneous fat (aged skin), reduction in organ size, and impaired hair growth^34,67^. Surprisingly, CR decreases survival in *Bmal1*^*−/−*^ mice, while failing to reduce IGF-1 and insulin levels^68^ or to improve circadian rhythmicity of clock genes^69^. It is unclear whether this is a specific effect of BMAL1, or whether CR also has detrimental effects in other clock mutant mice.

Tissue-specific responses to CR add an extra layer of complexity in understanding the relative contributions of nutrient-sensing pathways to longevity. For example, activation of SIRT1 has been proposed to mediate the effects of CR on lifespan. Yet, although CR increases SIRT1 in most tissues, it decreases it in liver^70^. Moreover, while brain-specific expression of SIRT1 is sufficient to extend lifespan in mice^71^, whole-body overexpression of SIRT1 has no such beneficial effect^72^. Experimental disruption of mTOR signaling in mice also has tissue-specific effects: in adipose tissue mTOR inhibition is beneficial, but in muscle it is deleterious^73^. A recent study showed that tissue-specific nutritional memory impairs the full benefits of CR if applied late in life^74^. Switching 24-month-old female mice from ad lib to CR slightly increases lifespan as compared to ad lib controls, but does not reach the lifespan extension observed in the life-long CR cohort^74^. Genome-wide comparisons revealed that while the liver was mostly reprogrammed by CR, ~13% of the genes in the white adipose tissue involved in lipogenesis and inflammation, were unresponsive to late-onset CR^74^. Therefore, many questions remain about the beneficial effects of CR in different tissues, in which tissue-specific circadian rhythms may play a significant role (Fig. 2 and Supplementary Table 1).

Two independent studies demonstrated that CR improves metabolic fitness and healthspan in rhesus monkeys, but reported a discrepancy on longevity, with either no effect^75^ or improvement^76^ with CR. Survival results were reconciled by considering differences in feeding behavior, age of treatment, genetic background, and other variables, and concluded that CR improves longevity in non-human primates^77^. The timing of food access partly explains the discrepancy in survival: CR had no effect on survival in the study that allowed the control group to eat ad lib, but only during the daytime, as food was removed every night^77^. This study was later replicated in mice, in which animals eating a single meal ad lib with variable periods of fasting lived longer than mice with 24 h food access^49^. It remains unknown whether these mechanisms also apply to humans.

A major confounding aspect of most CR regimens is that they change both the amount of food eaten and the temporal pattern of food intake^23^. CR protocols are often constrained by human schedules, with food provided in the morning when nocturnal rodents would not normally eat. With limited food access, rodents tend to eat their allotment as soon as the food is available, thereby self-imposing daily cycles of feeding and fasting^63^. Because food intake synchronizes metabolic function and hormone production throughout the body, the timing of food intake is critical. An intriguing study found no differences in lifespan in calorically restricted female mice with access to a single meal during the daytime, a single meal at night, or 6 meals at night^78^, suggesting that long-term effects of CR could be independent of feeding time. However, an important consideration is that CR mice eat shortly after food is made available;^63^ which allows the animal to remain close to its normal nocturnal behavior (driven by the light/dark cycle) by extending the active phase for <2 h. Alternatively, perhaps day-fed and night-fed groups extend lifespan through different mechanisms, as revealed by changes body temperature rhythms with opposite 24 h profiles^78^. Further experiments are required to elucidate, which possibility accounts for these findings. Altogether, despite many studies using CR, the contribution of the timing of food access by itself has become a new area of exploration to unravel the antiaging mechanisms induced by CR.

### Time restriction

Environmental signals, including food, can act as powerful non-photic clock entraining agents^79^. When food access is restricted to a few hours in the daytime (when nocturnal animals normally eat only sparingly), rodents increase their activity anticipating the time of food availability^80,81^. Such unnatural TR paradigms also shift the phase of circadian rhythms by many hours in peripheral clocks such as the liver, without a significant effect on the SCN^80–83^. This internal desynchrony in the organism, with central and peripheral clocks out of phase, has been associated with metabolic disorder. However, a combination of TR with either CR or highly palatable food results in a stronger stimulus capable of resetting both central and peripheral clocks^84^. In traditional TR protocols, a large shift in the core transcriptional clock in the liver is accompanied by complex changes in phase and amplitude of clock-controlled genes^83^. Thus, it is clear that feeding/fasting cycles are powerful cues for peripheral circadian clocks, with both CR and TR reprograming the circadian profiles of the transcriptome and metabolome^27,65,66,81,83,85–88^.

Mice consume 75–85% of their calories at night (active phase), and high-fat diet (HFD) dramatically disrupts this feeding pattern by increasing ~50% of intake during the rest phase^89^. Such diet-induced disruption in feeding behavior contributes to the development of obesity and diabetes in mice given HFD. Indeed, eating exclusively at the wrong time of the day increases weight gain and susceptibility to obesity, diabetes, and cardiovascular disease, while also negatively impacting learning and memory^85–87,90^. Conversely, restricting food intake to the active phase protects against HFD-induced obesity, hepatic steatosis, hyperinsulinemia, and inflammation^85,90^. While HFD dampens circadian rhythms of metabolic genes^89^, limiting food during the night-time restores the proper oscillations and prevents metabolic syndrome in WT^85,87^ and arrhythmic clock mutant mice^91^. Despite the short-term metabolic benefits, the impact of TR on longevity remains to be determined. Because TR is difficult to maintain long term, automated feeders may prove valuable in overcoming methodological limitations^63^.

### Fasting

Fasting is the most extreme example of dietary restriction. Chronic intermittent fasting (IF) involves alternating 24 h of minimal food intake with 24 h of unrestricted intake, or cycles of periodic fasting (PF) for several days^46^. In rodents, IF extends lifespan, protects against obesity, cardiovascular disease, hypertension, diabetes, and neurodegenerative diseases^92^. IF improves overall health in aged animals by inducing autophagy^93^ and thermoregulation^94^ while enhancing glucose metabolism^95^ and promoting neuronal resistance to injury independently of weight loss and caloric intake^96^. Importantly, the effects of IF upon body weight and lifespan depend on genotype, strain, and age of treatment^97^. GH/IGF-1 signaling seems to be a key mediator of the effects of IF, since mice lacking GH receptor fail to respond to IF^98^. Interestingly, IF alters the phase of circadian genes in the liver^99^. However, the long-term effects of IF on central and peripheral clocks are unknown.

Fasting periods between meals extend lifespan in mice, regardless of caloric intake and food composition^49^. When provided one single meal per day (MF) matching ad lib intake, mice have variable fasting intervals. However, even with food starting during the rest phase (3 h before lights off), MF mice live longer than mice with 24 h food access. Within the MF group, it would be interesting to determine whether longer fasting periods correlate with increased survival. Given individual differences in food intake (both amount and feeding pattern), recording the unrestricted ad lib feeding pattern as a baseline for each mouse would be useful for determining to what extent each imposed feeding protocol (CR, TR, or IF) also affects other feeding parameters. Thus, further studies are required to disentangle the contributions of the amount and the timing of food intake on survival.

A fasting-mimicking diet (FMD) has been developed to confer similar benefits of prolonged fasting, without the burden of several days of starvation. FMD is a low-cal/low-protein diet, with plant base ingredients. In mice, FMD enhances cognitive function, reduces adiposity, ameliorates bone loss, reduces the incidence of neoplasia, and boosts the immune system, but does not prolong maximum lifespan^100,101^. FMD restores insulin secretion from pancreatic islets from Type 1 diabetic (T1D) patients and reverses both T1D and T2D phenotypes in mouse models. Also, it decreases circulating levels of IGF-1, insulin, and glucose, while increasing plasma concentrations of IGF-binding protein 1 and ketone bodies and increases the number of progenitor stem cells^102^. Therefore, this approach of mimicking fasting without actually fasting effectively prolongs healthspan. It would be interesting to explore whether FMD, given for a few days every 2–3 months, enhances healthspan by resetting internal clocks or restoring metabolic rhythms.

### Changing food composition

Dietary restriction is extremely difficult to sustain long term, and compliance often decreases over time. As an alternative, several groups have explored reducing specific dietary components, rather than decreases in overall food intake. Two examples of modifications in dietary composition are ketogenic diets (KD) and protein restrictions (PR).

Ketogenic diets (KD) are low-carb, high-fat diets that recapitulate certain metabolic aspects of dietary restriction, such as reliance on fatty acid metabolism and production of ketone bodies as an energy source. In mice, KD improves healthspan, slows down age-associated neurological decline, and increases median, but not maximum lifespan^103,104^. Interestingly, KD reshapes liver and gut clocks^105^ increasing chromatin recruitment of BMAL1 and promoting higher amplitude rhythms in the liver, along with increasing rhythmicity in serum ketone bodies and PPARα target genes in the gut. Unlike HFD, which is rich in both in fat and carbohydrates, KD does not change feeding patterns, as mice continue eating mainly at night-time. The maintenance of rhythmic behavior with KD likely enhances tissue-specific rhythms and contributes to improving overall health status.

Limiting proteins or certain amino acids in the diet can also reduce the incidence of age-associated diseases and simultaneously increase lifespan^106^. Protein restriction increases a fasting-induced hepatokine, FGF-21, in rodents and humans^107^. In mice, overexpression of FGF-21 has been reported to extend lifespan by inhibiting GH/IGF-1 signaling pathways in the liver^108^. These FGF-21 overexpressing mice not only have elevated, but also highly rhythmic, levels of circulating FGF-21 that modulate circadian behavior^109^. In addition to PR, methionine restriction (MR) extends health and lifespan in mouse models of normal^110^ and accelerated aging^111^ as a consequence of reduced inflammation and increased autophagy and DNA stability. In contrast with these restrictive diets, which are difficult to implement long term, glycine supplementation mimics MR and promotes lifespan in rodents^112^. Glycine has also been implicated in regulating aspects of circadian biology. Glycine supplementation promotes sleep and hypothermia via NMDA receptors in the SCN in rats^113^ and it is able to synchronize SCN rhythms in vitro^114^. The mechanisms by which glycine acts on circadian clocks and how these cues are transmitted throughout the body are not yet fully understood. Taken together, these findings suggest that limiting specific dietary components is another viable alternative to improve metabolic function without constant CR. Further studies are required to establish whether restricting or supplementing at a specific optimal time of day could expand their benefits on healthspan and longevity.

## Implications for human health

### Caloric restriction and fasting-mimicking diets in humans

Long-term CR also improves several markers of health in humans, both in obese and non-obese individuals^115,116^. These include decreased body weight, metabolic rate, and oxidative damage^117^, lower incidences of cardiovascular disease^118^ and cancer;^119^ and decreased insulin-Akt-FOXO signaling pathway activity^120^. A new multicenter trial called the CALERIE, is currently assessing different aspects of human physiology as a consequence of long-term CR in over 200 individuals^121,122^. Even in clinical trials, long-term compliance with CR is low, and often only a partial goal of food restriction is achieved. However, highly motivated individuals, such as those in the long-term CR with Optimal Nutrition (CRONies) study, as well as observational studies of centenarians in Okinawa, Japan who have had CR for most of their lives^115,118^, provide evidence that CR can have benefits in humans.

Several short-term clinical trials have shown that alternate-day fasting can deliver benefits similar to CR in terms of weight loss and cardiometabolic health, including reduction in body weight and improved lipid profiles, lower blood pressure, and increased insulin sensitivity^123–126^. Fasting also appears to have anti-cancer properties in humans^127^, and it increases susceptibility of cancers to certain chemotherapeutics^128^. A recent clinical trial tested the feasibility of incorporating short-term fasting (72 h fasting around the time when the therapeutic agent is administered) into platinum-based chemotherapy. Similar to what was observed in mice, short-term fasting prior to chemotherapy administration appeared to protect against toxicity^129^. Additionally, fasting during chemotherapy was well-tolerated and reduced hematological toxicity in HER2-negative stage II/III breast cancer patients^130^.Nevertheless, implementing alternate-day fasting or similar interventions can still be extremely difficult.

Other diets have also been tested in humans with sleep abnormalities. A ketogenic diet^131^ can normalize sleep patterns in association with a loss in body mass^132–134^. In healthy, non-obese, normal sleepers, a ketogenic diet increases slow-wave sleep and decreases REM sleep compared to a high-carbohydrate, low-fat diet^135^. Similar to methionine restriction in animal models, low methionine levels found in vegan and vegetarian diets also confer metabolic benefits by increasing circulating levels of FGF-21 in humans^136^.

### Time restriction

With increasingly sedentary lifestyles and 24/7 access to food and artificial light, the burden of chronic diseases has also grown. The detrimental effects of poor eating behaviors are not only consequences of unhealthy diet (high fat, high sugar, highly processed foods), but also the timing of when the food is consumed. More than half of the individuals in diet studies regularly eat over a period of 15 h or longer each day, fasting only while they sleep^137,138^. Interestingly, the timing of food intake relative to the natural increase in melatonin (which marks the beginning of night for each individual) is significantly associated with body fat percentage and body mass index^139^. Nonlean individuals consume most of their calories 1.1 h closer to melatonin onset than lean individuals, suggesting that the timing of food intake is also a key aspect of metabolism in humans.

In humans, as in mice, the timing of calorie consumption impacts metabolic status and body weight maintenance, even during a short-term feeding regimen. For example, in a 20-week dietary intervention in 420 obese and overweight individuals, the timing of the main meal (i.e., lunch for the Spanish population) was predictive of weight-loss success independent from total 24 h caloric intake^140^. Body weight and optimal metabolic activity also appear to depend on the time of day of the eating window. Implementing a time-restricted feeding schedule decreases body fat, fasting glucose and insulin levels, insulin resistance, hyperlipidemia, and inflammation in humans^141–143^. Remarkably, there are further benefits if the food is consumed earlier in the day. When a 6 h feeding period, with dinner before 3 PM was compared to a control schedule (12 h feeding period) for 5 weeks in a randomized crossover trial of prediabetic men, insulin sensitivity, blood pressure, and oxidative stress levels were improved during the early time-restricted feeding regimen without any effect on body weight^144^. Other studies have suggested that the additional benefits are not simply because early TR also increases the fasting period; instead, there appears to be an optimal time for food consumption. In fact, restricting food intake to the late afternoon or evening (after 4 PM; late TRF) either has no effect or worsens postprandial glucose levels, glucose tolerance, blood pressure, and lipid levels^141,143,145^. Furthermore, early dinner improved glucose tolerance especially for subjects carrying a diabetes risk allele *MTNR1B* (melatonin receptor)^146^, supporting links between circadian melatonin and glucose control in humans.

In a laboratory setting participants were given three meals (breakfast, lunch, dinner) at 5 h intervals, beginning either 0.5 (early) or 5.5 (late) hours after waking. Interestingly, plasma glucose rhythms were delayed by over 5 hours and average glucose concentrations decreased following the late feeding schedule. Moreover, *Per2* mRNA rhythms in adipose tissues were delayed by 1 h after 6 days of late feeding, indicating that human molecular clocks may be regulated by feeding time and could influence plasma glucose^147^. Similarly, another study showed that skipping breakfast influences the circadian rhythms of clock genes and their targets and is correlated with increased postprandial glycemic response in both healthy individuals and individuals with diabetes^148^. A recent randomized crossover trial elegantly showed that healthy patients exhibit lower glucose levels, reduced hunger, and increased diet-induced thermogenesis (amount of energy used for digestion and absorption of nutrients) when eating high caloric breakfasts as compared to high caloric dinners^149^. Taken together, these studies suggest that a restricted window of food intake early in the day is the most beneficial.

### Consequences of misalignment of the circadian system

Genetic polymorphisms make some people more likely to behave as an early bird (early chronotype) or a night owl (late chronotype). A late chronotype may be detrimental in our scheduled society, as these individuals typically experience a mild form of circadian misalignment^150^. This link between chronotype and misalignment was also suggested in a study of 163 prediabetic or type 2 diabetic individuals’ eating and sleeping patterns. This study found that, after adjusting for age, sex, BMI, and statin use, a late chronotype was associated with higher inflammation in diabetic individuals^151^.

A forced misalignment of these circadian rhythms with the environment, as happens in jet lag and shift work, is detrimental to health^152^, with increased risk of type 2 diabetes^153^ and cancer^154,155^. This has been shown in the laboratory, when, during an 8-day gradual misalignment protocol between behavioral cycles (fasting/feeding and sleep/wake cycles) and endogenous circadian cycles, 10 adults showed decreased leptin, increased glucose despite increased insulin, increased mean arterial pressure, and reduced sleep efficiency^156^. This 8-day misalignment was also sufficient for 30% of the subjects to exhibit postprandial glucose responses in the range typical of a prediabetic state.

In another study, 14 healthy lean young men were exposed to a more drastic 12-h-shift misalignment protocol, after which insulin sensitivity tests and skeletal muscle biopsies were performed. After 3.5 days of circadian misalignment, these individuals showed normal hepatic insulin sensitivity but muscle insulin resistance due to reduced mitochondrial function and nonoxidative glucose disposal in the muscle^157^. Molecular analysis of muscle biopsies revealed that the molecular circadian clock was not aligned to the inverted behavioral cycle, possibly because it would take longer than 3.5 days to entrain to the 12 h shift. Nonetheless, fatty acid metabolism and PPAR signaling were drasticallly increased from 7 AM to 7 PM in the misaligned condition, revealing the human PPAR pathway as a key player in disturbed energy metabolism with circadian misalignment^157^. Although this protocol was extreme, it showed that glucose tolerance in humans can be altered even after a short-term circadian misalignment in a laboratory setting^158^.

### Sleep restriction

In addition to the circadian aspects of sleep/wake cycles, the total amount of time spent sleeping also appears to have an impact on metabolism and health. More than 30% of adult men and women report fewer than 6 h of sleep per night, well below the National Sleep Foundation’s recommendations^159^. Patients with sleep disorders have altered glucose homeostasis and a higher risk for developing obesity. In healthy individuals, sleep deprivation is inversely correlated with body weight, suggesting that sleep deprivation plays a key role in regulating energy balance^160^. Total or partial sleep deprivation results in increased sympathetic nervous system activity, increased evening cortisol, and increased daytime GH levels^161,162^. After sleep restriction, leptin levels are lower and ghrelin levels are higher^163,164^. Indeed, a meta-analysis of 11 studies showed that after partial sleep deprivation energy intake was increased by ~385 kcal per subject^165^. Since there was no significant change in total energy expenditure or resting metabolic rate, sleep deprivation led to a positive energy balance, which in the long term may contribute to weight gain in these individuals. Importantly, sleep restriction not only increases energy intake but also extends the eating window and also increases the preference for frequent energy-rich snacks over regular meals^166^. Such altered patterns of food consumption likely contribute to the detrimental metabolic effects of sleep deprivation.

## Circadian medicine: optimizing when to treat

**Fig. 4:**
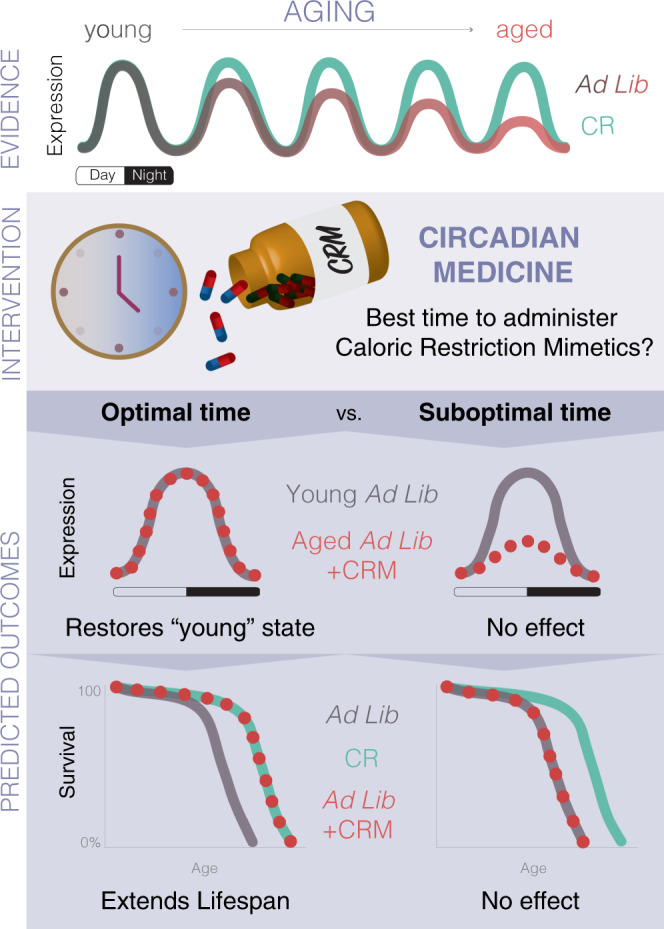
Model of how Circadian Medicine can be used as an optimized intervention to improve circadian rhythms and potentially promote lifespan. The top panel reflects the current evidence that, (1) aging-related pathways oscillate throughout the day; (2) circadian rhythms decline with age and restoring rhythms improves healthspan; and (3) CR, the most robust lifespan-extending intervention, remarkably protects against the age-dependent dampening of circadian rhythms. Circadian medicine introduces a time-of-day concept for administration of drugs. Considering most aging-related genes are circadian, perhaps there is an optimal time for interventions such as CR mimetics (CRMs). The hypothesis behind this model is that there is an optimal time to administer antiaging drugs, which can restore the proper rhythms targets, and consequently boost survival. If there were an optimal time, we would expect robust circadian rhythms even in an aged individual resembling a young state, potentially leading to lifespan extension. On the contrary, a suboptimal time of administration would not be effective or would require a higher dose to reach similar beneficial effect. Tailoring the treatment for each drug as to how often and what time of the day is still required, as it depends on pharmacokinetic properties, tissue-specific pathways, potential sex-differences, and other factors.

The National Institute of Aging Intervention Testing Program (ITP) is a multicenter initiative assessing treatments with the potential to extend lifespan and delay disease onset and dysfunction in mice^167^. Successful treatments include FDA-approved drugs to treat cancer and diabetes, such as rapamycin (mTOR inhibitor)^168,169^ and metformin (AMPK activator)^170,171^. These drugs are classified as caloric restriction mimetics (CRMs) since they target similar molecular pathways and confer the health benefits of CR without actually limiting food intake (Fig. 2) (thoroughly reviewed in ref. ^172^). Genome-wide studies with circadian sampling in rodents, non-human primates, and human tissues have highlighted that the targets of >80% of the FDA-approved drugs exhibit daily rhythms^57,173^. Thus, dosage timing may help to optimize benefits of antiaging drugs, especially when acting on oscillating or moving targets (Figs. 2, 4).

For practical reasons, most drugs tested in mice are supplemented in the diet or drinking water, which leads to voluntary night-time administration^174^. However, this may not be the best time to administer drugs whose targets have circadian rhythms of expression. Because numerous aspects of human physiology are under circadian control, there are windows of opportunity for interventions by simply administering drugs when their targets are at the right expression level to restore (Fig. 4). This is the basis of a concept known as Circadian Medicine, or Chronotherapy^173^ (Fig. 4). Circadian medicine as a model for antiaging interventions is based on current evidence that: (1) circadian rhythms decline with age^8,27–31,65,66,175^, (2) disruption of circadian rhythms leads to metabolic disorders^20,38–40,43,44,90^ and shortens lifespan^25,32–37,42^, while restoration of circadian rhythms promotes health^19,26,91^ and longevity^35–37,175^, and (3) aging-related pathways oscillate throughout the day^6–10,51,57^. Therefore, a plausible hypothesis is that there is an optimal time to administer drugs, which can restore the proper rhythms of targets, and ultimately result in lifespan extension (Fig. 4), while a suboptimal time would not have any benefit. This would require further tailoring treatments for each drug as to how often and what time of day it is needed, as it depends on pharmacokinetic properties, tissue-specific pathways, and potential sex-differences. Thus, moving forward, it will be necessary to develop new tools for administering interventions at specific times while controlling for frequency and amounts given. For example, it would be useful to develop an automated computer-controlled device to control access to drugs administered in the water or food, such as a gate alternating access between treated and nontreated water. For food-supplemented drugs, a separate compartment could be designed to deliver drugs as pills independently of the food dispenser. In addition, other promising approaches, such as mini-pumps, could be adapted for drug delivery.

There are many examples of treatments for aging-related diseases that are more effective when given at specific times of day, including cancer^176–178^, T2D^179^, and hypertension^180^. Aspirin extends lifespan in male mice^181^ and is used as a secondary prevention for cardiovascular disease (CVD) in humans^182^. The efficacy and outcome of aspirin treatment highly depends on dosing timing^183–188^. In humans, a randomized crossover trial showed that aspirin reduces blood clotting more effectively when taken at bedtime rather than in the morning^186^. Conversely, increased hemorrhage, risk of CVD and all-cause mortality was found in a placebo-controlled trial with healthy older adults (65+) receiving aspirin during breakfast^187,188^.

Polyamines are an interesting success story highlighting how a pharmacological intervention could mediate crosstalk between circadian clocks, metabolic pathways, and lifespan. Polyamines (e.g., putrescine, spermidine and spermine) are involved in cell growth, survival, and proliferation^189^. Despite an association of increased levels with cancer, both spermidine supplementation and high-polyamine diets increase lifespan in rodents^189^. Interestingly, polyamines show circadian oscillations and also influence circadian periodicity by regulating PER2/CRY1 interactions^175^. Remarkably, polyamine oscillations exhibit an age-dependent lengthening of period that can be reversed by dietary supplementation of polyamines in drinking water^175^.

Other potential drugs for delaying age-related diseases are those that target endogenous clocks. Screens to identify small molecules that modulate the clock have indeed proven useful at identifying clock-enhancing drugs^190,191^. One such molecule, a natural flavonoid compound called nobiletin (NOB) was found to mitigate body weight gain without altering food intake, stimulate energy expenditure and circadian activity, enhance glucose and insulin tolerance, diminish lipid content, and improve mitochondrial respiration in mice^26,192^. This study provided clear evidence that maintenance of a robust circadian organization within the organism protects against metabolic disruption.

### Methods to infer individual internal timing

Internal circadian time varies among individuals, as it is influenced by many factors, including work schedules, feeding regimens, genetic predisposition, age, sex, environmental light levels, and seasons. Current efforts are dedicated for leveraging individual patient’s circadian clocks to personalize healthcare. Several algorithms have been developed to identify reliable markers of internal timing based on blood and brain transcriptomic datasets^193–200^. The first method developed to infer internal timing, called Molecular Timetable, is composed of ~100 time-indicating-genes identified from mouse liver microarray datasets^193^. Identifying circadian targets in humans has been challenging, since genome-wide datasets for most tissues rarely include time of day during which samples were collected. Some algorithms have been developed to this end: CYCLOPS that reveals human transcriptional rhythms in health and disease^198^; BodyTime, a simple assay to determine the internal timing of an individual from a single blood sample taken at any time during the day^199^; and more recently TimeSignature, a machine learning-based algorithm designed to accurately predict internal timing from blood samples (±2 h) using ~40 genes as predictor markers^200^. All of these provide promising tools for translational studies and individualized circadian medicine (Fig. 4).

In addition to individual variation in rhythms, the circadian system is highly amenable to resetting signals, including environmental changes (light/dark cycle, food availability), behavior (sleep, exercise, feeding), endogenous metabolites, and hormonal status. Moreover, pharmacological interventions to extend longevity in mice often exhibit sexual dimorphism^201–203^ and depend at what age the treatment starts^112^, and thus are important factors to consider in addition to the timing of antiaging therapies. Incorporating these variables in assessing individual internal timing pushes the need for developing novel computational tools.

## Final remarks

As the human population ages, the increased risk of chronic diseases has become a public health burden worldwide. Additionally, ~39% of the world adult population is overweight due to unrestricted access to food and sedentary lifestyle, increasing the incidence of cardiovascular disease, obesity, diabetes, and stroke. Dietary interventions, including regulation of the amount and timing of food intake and length of fasting periods, have become attractive methods for mitigating age-related physical decline and chronic diseases. Although the specific mechanisms are far from being fully understood, a periodic break in energy intake appears to improve multiple risk factors and, in some cases, reverse disease progression in mice and humans. Going forward, it will be important to elucidate to what degree the effects of caloric restriction regimes are due to calories, fasting, and feeding time. In addition, pharmacological interventions targeting pathways improved by DR have become promising alternatives to restricted diets. Understanding how metabolic pathways change throughout the day may provide insights into when and how often treatments should be applied in order to minimize drug resistance and side effects (Fig. 4). Additionally, systematic studies are required to determine at what age treatment can be applied to provide maximum benefits. Integrating tissue-specific circadian oscillations in these pathways could also prove critical for pinpointing the optimal time to administer interventions in order to promote healthy aging.

## Supplementary information

Supplementary Information
