# Supplementary material for: Importance of circadian timing for aging and longevity
Source: Nat Commun. 2021 May 17;12:2862. doi: 10.1038/s41467-021-22922-6 (PMC8129076; doi:10.1038/s41467-021-22922-6)
Supplement: Supplementary file 1 — Supplementary Information [file 41467_2021_22922_MOESM1_ESM.pdf]

Common name	GeneID	Adrenal gland			Aorta			Brain			brain stem			BAT			Cerebelum			Distal Colon			Heart			Hypothalamus			Kidney			Liver			Lung			Muscle			Pituitary			SCN			Spleen			WAT		
		Mo	Ba	Hu	Mo	Ba	Hu	Mo	Ba	Hu	Mo	Ba	Hu	Mo	Ba	Hu	Mo	Ba	Hu	Mo	Ba	Hu	Mo	Ba	Hu	Mo	Ba	Hu	Mo	Ba	Hu	Mo	Ba	Hu	Mo	Ba	Hu	Mo	Ba	Hu	Mo	Ba	Hu	Mo	Ba	Hu	Mo	Ba	Hu	Mo	Ba	Hu
GH	Gh	n	n		n	n					n			n			n	n		y			y	n		n	n		n	n		n	n		n	n		n	n		n	n		y	n		n	n		n	n	
IGF-1	Igf1	n	n		y	n	n				y			y			n	n		n		n	y	n	n	n	n		y	n		y	n	n	y	n	n	n	n		y	n		y	n		n	n		n	n	y
PI3K	Pi3kca	n	n		y	n	n				n			n			n	n		y		n	y	n	n	n	n		y	n		y	n	n	n	n	y	y	n		n	n	n	n	n		n	n		y	n	n
	Pi3kcb	n	n		y	n	n				n			y			n	n		n		n	n	y	n	n	n		n	n		y	n	n	n	n	n	n	y		n	n	n	n	n		n	n		n	n	y
	Pi3kcd	n	n		n	n	y				n			n			n	n		y		n	y	n	n	n	n		n	n		n	n	n	y	n	n	n	n		n	n	y	n	n		n	n		n	n	n
	Pi3kcg	n	y		n	n					n			n			n	n		y			n	n		n	n		n	y		n	n		n	y	n	y	n		n	n		y	n		n	n		n	n	
AKT	Akt1	y	n		y	n	n				n			y			n	n		y		n	y	y	n	n	n		n	n		y	n	n	y	n	n	n	y		n	n	n	n	n		n	n		n	y	n
	Akt2	y	n		y	n	y				n			y			y	n		y		n	y	y	y	y	n		n	n		y	n	n	y	n	y	n	y		n	n	n	n	n		n	n		y	n	y
	Akt3	y	n		n	n	n				n			y			n	n		y		n	y	n	n	n	n		y	n		n	n	n	y	n	n	n	n		y	n	y	n	n		n	n		n	n	n
mTOR	Mtor	n	n		y	n	y				n			y			n	n		y		n	y	n	n	n	y		y	n		y	n	n	n	n	n	n	n		n	n	n	y	n		n	n		n	n	n
RICTOR	Rictor (TOR2 complex)	n	n		n	n	y				n			n			y	n		y		y	n	n	y	n	n		n	n		y	n	y	n	n	y	n	n		y	n	n	y	n		n	n		n	n	y
RPTOR	Rptor (TOR1 complex)	n	n		n	n	n				y			n			n	n		y		n	n	n	y	n	y		n	y		n	n	n	y	n	n	n	n		n	n	n	n	n		n	n		n	n	y
PTEN	Pten	n	n		y	n	n				n			n			n	n		y		n	y	y	n	y	n		y	n		n	n	y	y	n	y	n	n		y	n	n	y	n		y	n		n	n	n
FOXO	Foxo1	n	n		n	n	n				n			y			n	n		y		y	y	n	y	n	n		n	n		y	n	n	n	n	y	y	n		n	n	n	n	n		n	n		n	n	n
	Foxo3	n	n		n	n	n				n			n			n	n		y		n	y	n	n	n	y		y	n		y	n	y	y	n	y	n	n		n	n	n	y	n		n	n		n	y	y
	Foxo4	n	n		n	n	n				n			y			n	n		n		n	n	n	n	n	n		n	n		n	n	n	n	n	y	y	y		n	y	n	y	n		n	n		n	n	y
	Foxo6	n			n						n			n			n			n			n			n			n			n			n			n			n			n			n			n		
AMPK	Prkaa1	n	n		y	n	n				n			y			n	n		y		y	y	n	n	n	y		n	n		n	n	n	n	n	n	n	n		n	n	n	y	n		n	n		y	n	n
	Prkaa2	y	n		n	n	y				n			y			n	n		y		y	n	n	y	n	y		y	n		y	n	y	y	n	n	n	y		n	n	n	y	n		n	n		n	n	y
SIRT	Sirt1	n	n		n	n	n				n			n			n	n		y		n	n	n	y	n	y		n	n		y	n	n	y	n	y	n	n		n	n	n	y	n		n	n		n	n	n
	Sirt2	n	y		n	n	n				n			n			n	n		y		y	n	n	y	y	n		n	n		y	n	n	y	n	n	n	y		n	n	n	n	n		n	n		n	n	n
	Sirt3	y	n		y	n	y				n			y			n	n		n		n	y	n	y	n	n		y	n		y	n	n	n	n	y	n	n		n	n	n	y	n		n	n		n	y	y
	Sirt4	n	n		n	n					n			n			y	n		y		y	n	y	y	n	n		n	n		y	n	n	n	n	n	n	n		n	n	n	n	n		n	n		n	n	y
	Sirt5	n	n		n	n	n				n			n			y	n		n		n	y	n	y	n	n		n	n		y	n	y	n	n	n	n	n		n	n	y	n	n		n	n		n	n	y
	Sirt6	n	n		n	n	n				n			n			n	n		n		n	n	n	n	n	n		n	y		n	n	y	n	n	n	n	n		n	n	n	n	n		n	n		n	y	n
	Sirt7	n	n		n	n	n				n			y			n	n		y		n	y	n	n	n	n		n	n		y	n	n	n	n	n	n	n		n	n	n	n	y		n	y		n	n	n
PGC-1a	Ppargc1a	y	y		y	n					n			y			n	n		n		y	y	n	y	n	n		n	n		y	n	n	y	n	n	n	n		n	n	y	n	n		n	n		n	n	n
FGF-1	Fgf1	n	n		y	n	n				n			y			n	n		y		y	n	n	n	n	n		y	n		y	n		y	n		n	n		y	n	n	y	n		n	n		n	n	n
FGF-21	Fgf21	n	n		n	n					n			n			n	n		y			n	n		n	n		n	n		y	n	y	n	n		n	n		n	n		n	n		n	n		n	n	
PARP-1	Parp1	n	n		n	n	y				n			n			n	n		n		n	y	n	n	n	n		n	n		y	n	n	n	n	y	n	n		n	n	n	n	n		n	n		n	n	n

Supplementary Table 1. Tissue-specific circadian oscillations of aging-related genes. Nutrient-sensing genes associated with longevity, undergo circadian oscillations in several mammalian tissues; including mice, baboons and humans. Aging-related genes expressed in a circadian manner in mouse (purple), baboon (salmon) and human (green). Genes that are non-circadian in each tissue are represented in grey. White color indicates no data. RNA-seq and microarray containing circadian datasets for mouse and other tissues extracted from CircaDB (http://circadb.hogeneschlab.org, Hogenesch laboratory).

Legends

n	not cycling
y	circadian in Mouse
y	circadian in Baboons
y	circadian in Humans
	no data available/reported

Definition of "**circadian**" expression when JTK-cycle (circadian algorithm) p-value <0.05

References

CircaDB: http://circadb.hogeneschlab.org/

Koike, N., *et al.* Transcriptional architecture and chromatin landscape of the core circadian clock in mammals. *Science* **338**, 349-354 (2012).

Zhang, R., Lahens, N.F., Ballance, H.I., Hughes, M.E. & Hogenesch, J.B. A circadian gene expression atlas in mammals: implications for biology and medicine. *Proc Natl Acad Sci U S A* **111**, 16219-16224 (2014).

Mure, L.S., et al. Diurnal transcriptome atlas of a primate across major neural and peripheral tissues. *Science* **359**, eaao0318 (2018).
